# Evolution of Pore Structure and Meso-Damage Simulation of Aeolian Sand Self-Compacting Concrete Under Freeze–Thaw Cycles

**DOI:** 10.3390/ma19132830

**Published:** 2026-07-02

**Authors:** Xin Tong, Qing Liu, Fengxia Han, Huidong Liu, Guochao Huang

**Affiliations:** 1 College of Civil Engineering and Architecture, Xinjiang University, Urumqi 830017, China; 107552304726@stu.xju.edu.cn; 2 Key Laboratory of Building Structure and Seismic Resistance of Xinjiang, Urumqi 830017, China; fxhan@xju.edu.cn; 3 China Construction Third Engineering Bureau Group Co., Ltd., Xinjiang Branch, Urumqi 830028, China; huidongliu2026@163.com; 4 Urumqi Cheng’erxin Engineering Testing Co., Ltd., Urumqi 830022, China; guochaohuang2026@163.com

**Keywords:** aeolian sand self-compacting concrete (ASSCC), CT scanning technology, pore structure evolution, freeze–thaw cycles, damage mechanism

## Abstract

Currently, existing studies primarily perform damage simulations based on random aggregate mesoscale models of concrete. In contrast, research on freeze–thaw numerical simulations based on realistic concrete mesostructural models remains relatively scarce. In this study, based on X-ray computed tomography (CT) scanning technology, the influence of freeze–thaw action on the pore structure evolution law of aeolian sand self-compacting concrete (ASSCC) was analyzed. Mesoscale characteristics of the mortar, aggregates, and pores were extracted using image processing software, and a realistic mesostructural model of ASSCC was subsequently established. Furthermore, numerical simulations of the freeze–thaw cycle process were conducted using the finite element software ABAQUS. The results indicated that during the initial freeze–thaw stage, the formation of small new pores predominated within the concrete. As the freezing and thawing cycles progressed, these pores gradually interconnected and coalesced into larger irregular pores, which eventually led to the development of penetrating cracks that resulted in structural failure of the ASSCC. The mesostructural model derived from CT data effectively simulated the failure patterns and mechanical performance of ASSCC under both uniaxial compression and freeze–thaw conditions. This provides an effective means for predicting the mechanical properties of concrete under freeze–thaw cycling conditions.

## 1. Introduction

Self-compacting concrete (SCC), a high-performance concrete, is widely used in construction due to its excellent flowability, compactness, stability, and compressive strength. Its ability to eliminate the need for vibration during pouring enhances construction efficiency [1]. In northwestern China, the high transportation costs of conventional river sand significantly increase the overall cost of concrete production. Given the abundant aeolian sand resources in this region, replacing traditional river sand with aeolian sand as fine aggregate for SCC not only alleviates the shortage of river sand resources and reduces transportation costs but also aligns with the concept of green and low-carbon construction. Studies [2,3,4,5] have demonstrated that an appropriate substitution ratio of aeolian sand and a well-optimized particle size distribution can effectively fill internal pores within the concrete matrix. Furthermore, reactive components in aeolian sand can participate in pozzolanic reactions with cement hydration products (e.g., Ca(OH)_2_) to produce high-strength calcium silicate hydrate (C-S-H) gels. These processes enhance concrete density, thereby further improving its workability, mechanical properties, and durability.

Freeze–thaw damage is a critical factor influencing the durability of concrete structures in cold regions, and this damage is closely related to the meso-structure of concrete [6,7]. In recent years, CT scanning technology has emerged as an essential tool for capturing mesostructural characteristics and identifying defects in concrete [8,9,10,11,12]. Based on CT data, Li Y et al. [13] observed that freeze–thaw damage progresses from the surface inward in a spalling manner. They developed a damage model for the elastic modulus of cement mortar under salt-freezing conditions, incorporating mass loss and porosity, which showed good agreement with experimental results. Li N et al. [14] utilized CT scanning to monitor concrete damage during uniaxial compression, using CT-number-based damage variables and Poisson’s ratio to evaluate degradation. Zhao D et al. [15] compared the pore structures of rapid-hardening and ordinary foam concrete via CT image analysis, linking mesostructural parameters to their divergent early-age mechanical performance. Similarly, Wang R et al. [16] analyzed crack evolution and underlying mechanisms by combining CT imaging with crack statistics. Furthermore, with advances in computational technology, the finite element method (FEM) has been increasingly employed in recent years to simulate concrete damage processes, providing effective predictions of experimental outcomes and saving time and resources [17,18,19,20]. Wang R et al. [21] developed a two-dimensional mesoscopic model of concrete using Python and ABAQUS. They established a constitutive model for freeze–thaw damage, with “relative compressive strength” as the key variable and employed it to simulate compressive tests of concrete after freeze–thaw cycles. Peng R et al. [22] developed a three-dimensional stochastic aggregate model and performed numerical simulations of cracking in concrete under freeze–thaw cycles using a user subroutine in ABAQUS, effectively capturing the stress–strain evolution. Zhang M et al. [23] developed a 3D mesoscale concrete model accounting for the random shape and size of aggregates and used it to compare the uniaxial compressive failure mechanisms, modes, and stress–strain curves before and after freeze–thaw cycles, which validated the reliability of their modeling approach and parameters. Zhou S et al. [24] conceptualized concrete as a mesoscopic multiphase composite material consisting of aggregates, mortar, and pores, and established a mesoscopic model based on CT images for uniaxial compression simulation. A good agreement between the simulation results and the experimental data confirmed the reliability of the mesoscopic model. However, most existing studies have predominantly focused on characterizing freeze–thaw damage using mesoscopic parameters extracted from CT images or on damage simulation using randomly generated aggregate models. In contrast, numerical simulations of freeze–thaw cycles that incorporate realistic mesostructures remain scarce.

This study aimed to construct a mesoscopic ASSCC model based on CT images to investigate freeze–thaw-induced damage from a mesoscopic perspective. It also analyzed the evolution of internal pore structures in ASSCC after freeze–thaw cycles. ABAQUS23 software was employed to simulate freeze–thaw damage, and the simulation results were compared with experimental results to validate the model’s reliability. This approach provides a new framework for predicting freeze–thaw damage in ASSCC. Furthermore, this research offers a valuable reference for examining the interaction among the internal structure, mesoscopic damage, and macroscopic fracture failure of concrete.

## 2. Experimental Overview and Methodology

### 2.1. Raw Materials

**Figure 1:**
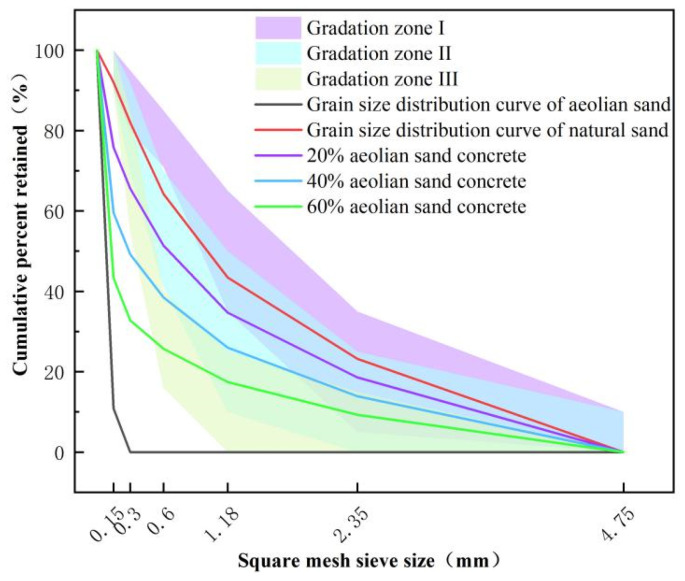
Gradation curves of aeolian sand and natural sand.

The test materials used in this study included Tianshan brand P·O 42.5 ordinary Portland cement, Class II fly ash and a Polycarboxylate-based high-performance water reducer with a water-reducing efficiency of 25–30%. Additionally, coarse aggregate with a particle size range of 4.75–19 mm and natural sand with an apparent density of 2863 kg/m^3^ were sourced from the Urumqi Western Construction Sand and Gravel Factory (Urumqi, China). Aeolian sand with an apparent density of 2658 kg/m^3^ was collected from the Gurbantünggüt Desert. Its particle size distribution curve is presented in Figure 1. The natural sand used in the test fell within Zone II of the gradation zones. This observation meets the requirement specified in Section 3.1.2 of the standard for quality and test methods of sand and crushed stone (or gravel) for ordinary concrete (JGJ 52-2006) [25], which states that “Zone II sand should be preferentially selected for concrete preparation.” Due to the aeolian sand’s poor particle size distribution, which falls outside the standard grading zone for sand, the experimental study employed an appropriate incorporation ratio of aeolian sand. The particle size distributions of fine aggregates with varying aeolian sand contents are shown in Figure 1. Local tap water from Urumqi City was used for the tests.

### 2.2. Mix Proportion

Based on the experimental data of the research team and existing studies [26,27], the Specification for Mix Proportion Design of Ordinary Concrete (JGJ 55-2011) [28], and the Technical Specification for Application of Self-compacting Concrete (JGJ/T 283-2012) [29], an aeolian sand incorporation ratio of 20% was selected. The specific mix proportions are presented in Table 1, where ASSCC denotes aeolian sand self-compacting concrete.

### 2.3. Experimental Methods

#### 2.3.1. Freeze–Thaw Cycle Tests

The freeze–thaw cycle test was conducted in accordance with the “rapid freeze–thaw method” specified in the Standard for Test Methods of Long-Term Performance and Durability of Ordinary Concrete (GB/T 50082-2024) [30]. A TDRF-1AF type freeze–thaw testing apparatus was employed for the experiment. The test specimens were divided into 10 groups, with each group containing three prismatic specimens measuring 100 mm × 100 mm × 400 mm. The specimens were subjected to 0, 25, 50, 75, 100, 125, 150, 175, and 200 freeze–thaw cycles, respectively. The freezing phase maintained a temperature of −18 °C for approximately 1.5 to 2.5 h, while the thawing phase kept the temperature at 5 °C for about 1 to 1.5 h. The transition duration between freezing and thawing phases was controlled to not exceed 10 min. Uniaxial compression tests were performed on 100 mm × 100 mm × 100 mm specimens obtained by cutting parent prisms after every 25 freeze–thaw cycles.

#### 2.3.2. CT Scanning Test

**Figure 2:**
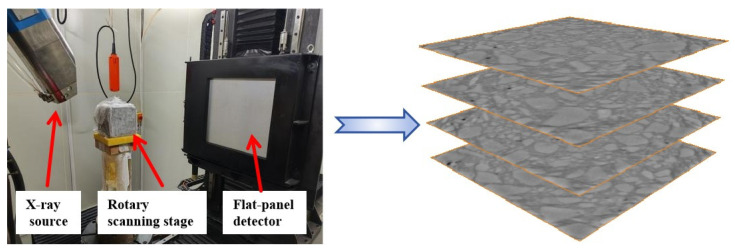
CT scanning system.

The test was conducted using a fifth-generation industrial CT system, specifically a cone-beam CT scanner, as illustrated in Figure 2. The system primarily consists of three core components: an X-ray source, a rotary scanning stage, and a flat-panel detector. The key operational parameters were configured as follows: voltage = 580 kV, current = 1.0 mA, exposure time = 500 ms, and a full 360° rotation. At 0, 100, and 200 freeze–thaw cycles, a specific ASSCC specimen was scanned using CT to observe the evolution of its internal damage.

## 3. Development and Validation of an ASSCC Mesoscopic Model

### 3.1. 3D Reconstruction of a Mesoscopic Model

#### 3.1.1. Model Development

**Figure 3:**
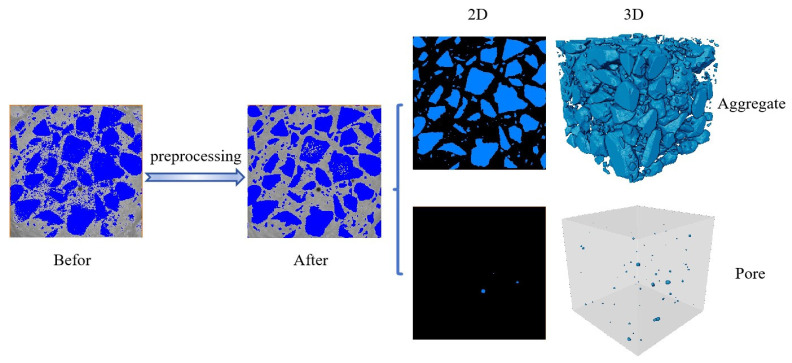
Model development.

The raw CT images were subjected to a preprocessing procedure to obtain high-precision mesoscopic structural features of ASSCC. First, a 3 × 3 median filter was applied to reduce noise while preserving essential image details. Subsequently, a background correction algorithm was implemented to eliminate gray-level gradients caused by non-uniform X-ray intensity. This operation significantly improved image quality and homogeneity. As illustrated in Figure 3, the preprocessed images following threshold segmentation exhibited clear phase interfaces and well-preserved aggregate morphology. The preprocessing procedure notably improved the signal-to-noise ratio (SNR) of the images, which provides a reliable foundation for subsequent quantitative mesoscopic analysis.

Threshold segmentation was performed on the preprocessed CT images using Avizo2022 software. The grayscale thresholds were set in the range of 0–35,000 for pores and 43,940–50,000 for aggregates. Manual corrections were required to rectify segmentation inaccuracies in certain localized regions. Finally, the aggregate and pore binary images were obtained through threshold segmentation, and a corresponding three-dimensional model was generated accordingly.

#### 3.1.2. Model Optimization

To improve the computational efficiency of the finite element model, geometric simplifications were applied to the aggregate model. Specifically, (1) aggregate particles smaller than 5 mm were removed, and (2) sharp edges and corners on the aggregate surfaces were smoothed. These simplification operations were performed using 3-matic21 software, and a sensitivity analysis was conducted to evaluate the impact of the simplification parameter Geometric Error (i.e., the maximum allowable geometric deviation introduced during the reduction of surface meshes).

**Figure 4:**
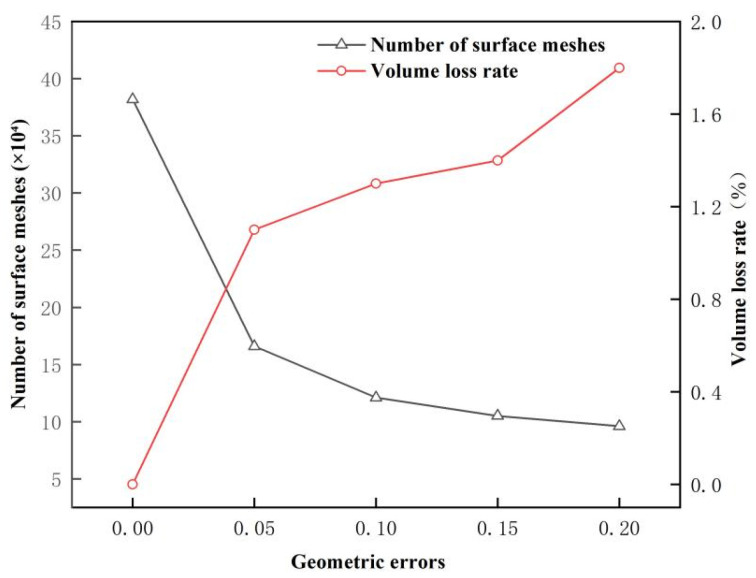
Sensitivity analysis of aggregate model simplification parameters.

As shown in Figure 4, an increase in Geometric Error resulted in a gradual rise in the aggregate volume loss rate. Simultaneously, the number of surface meshes in the aggregate model decreased sharply and eventually stabilized at approximately 100,000. To achieve a balance between computational efficiency and geometric accuracy, a Geometric Error of 0.15 was ultimately selected for the aggregate model simplification. The original aggregate model contained an extensive number of triangular facets (382,000), which posed significant computational challenges. After optimization, the number of facets was reduced to 98,000, representing a reduction of 72.5%. Notably, the surface morphology of the aggregates was effectively preserved, while the volume error was controlled within 2%. This ensured that the geometric accuracy of the simplified model met the requirements for reliable mechanical simulation.

**Figure 5:**
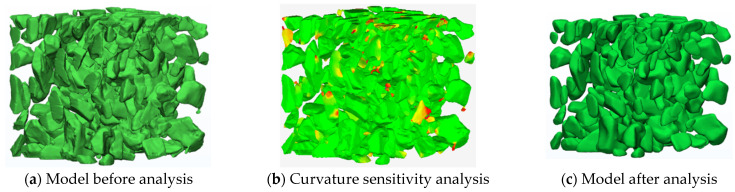
Curvature sensitivity analysis of aggregate model.

Despite the simplification, the aggregate model still exhibited defects such as sharp edges and grooves. A curvature sensitivity analysis was performed, and regions with excessively high curvature were smoothed. The processing steps are illustrated in Figure 5.

#### 3.1.3. Model Assembly

**Figure 6:**
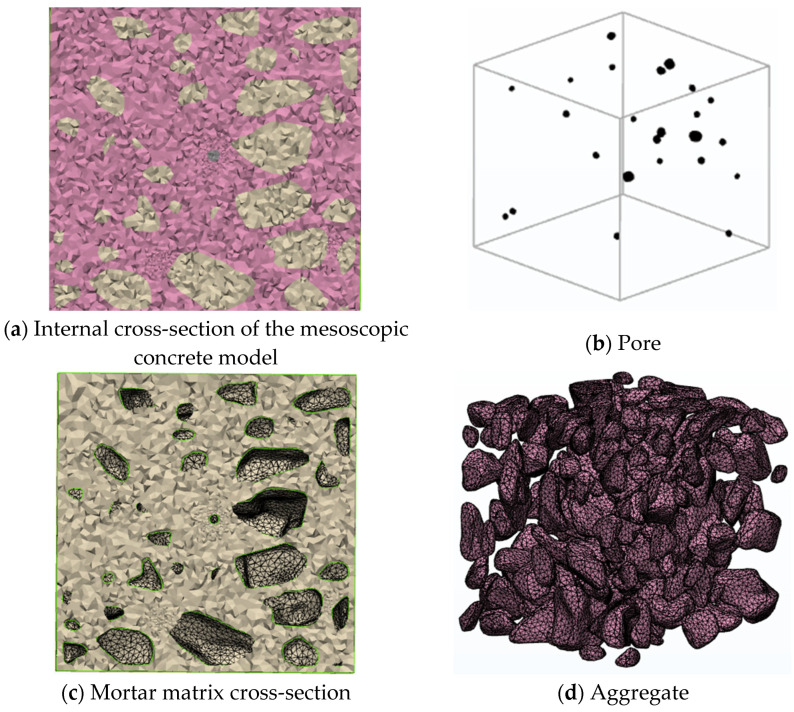
Mesoscopic concrete model.

A cubic specimen model with dimensions of 100 mm × 100 mm × 100 mm was created using 3-matic software. The simplified aggregate and pore models were imported into the cubic matrix and positioned accordingly. Boolean operations were performed to subtract the volumes of the aggregates and pores from the cubic matrix, thereby generating the mortar phase model. Subsequently, a non-manifold assembly was applied to integrate the mortar, aggregate, and pore models. This operation ensured that nodes were shared at all material interfaces to maintain structural continuity and compatibility. The finalized mesoscopic concrete model is presented in Figure 6.

### 3.2. Material Properties of Constituent Phases and Loading Conditions

#### 3.2.1. Mechanical Properties of Materials

Since the natural coarse aggregates generally exhibit higher strength compared to the mortar and the interfacial transition zone (ITZ), they are unlikely to develop penetrating cracks that lead to fracture failure. Consequently, the plastic deformation of the coarse aggregate phase was not considered, and a linear elastic model was adopted to describe its behavior. Qiao et al. [31] stated that, when the coarse aggregate content was 40%, the compressive strength of the mortar was 0.954 times that of the concrete. The elastic modulus and tensile strength of the mortar can be derived based on their empirical relationships with the compressive strength [32], as expressed in Equations (1) and (2). The material parameters for each phase are summarized in Table 2.

(1) $$E_{m}=1000(7.7Inf_{\mathrm{cm}}-5.5)$$

(2) $$f_{tm}=1.4Inf_{cm}-1.5$$

where *E_m_* is the elastic modulus of mortar (GPa); *f_cm_* is the compressive strength of mortar (MPa); and *f_tm_* is the tensile strength of mortar (MPa).

The mortar phase was simulated using the Concrete Damaged Plasticity (CDP) model. The stress–strain relationship and key parameters for the CDP model were determined in accordance with the Code for Design of Concrete Structures (GB 50010-2015) [33] and the mechanical parameters of the mortar material listed in Table 2.

#### 3.2.2. Parameters for the Cohesive Zone Model at the Mortar–Aggregate Interface

External loading-induced failure in concrete typically initiates either at the mortar–aggregate ITZ or within the mortar. Cohesive elements were incorporated at the mortar–aggregate interfaces to enhance the accuracy of the mesoscopic concrete model. The chosen element type was the 6-node 3D cohesive element (COH3D6). A bilinear traction-separation law was applied to characterize the cracking behavior of the ITZ in the concrete.

The quadratic nominal stress criterion (QUADS) was selected as the initial damage criterion. Damage in the cohesive element was assumed to initiate when the nominal stress satisfied Equation (3):

(3) $${\left\{\frac{\langle t_{n}\rangle }{t_{n}^{0}}\right\}}^{2}+{\left\{\frac{t_{s}}{t_{s}^{0}}\right\}}^{2}=1$$

where $t_{n}^{0}$ and $t_{s}^{0}$ are the maximum nominal stresses of the interface element under pure tension and pure shear states, respectively; $t_{n}$ and $t_{s}$ are the normal and tangential components of the stress at the interface element, respectively;   is the Macaulay symbol.

The progression of damage in the mortar–aggregate cohesive element was described using the Benzeggagh–Kenane (B-K) fracture criterion, which is suitable for mixed-mode (I/II) fracture scenarios. This criterion is expressed as follows:

(4) $$G^{C}=G_{n}^{C}+\left(G_{s}^{C}-G_{n}^{C}\right){\left\{\frac{G_{s}}{G_{n}+G_{s}}\right\}}^{\eta }$$

where $G_{n}^{C}$ and $G_{s}^{C}$ are the mode I and mode II fracture energy of the cohesive element, respectively; $G_{n}$ and $G_{s}$ are the deformation energy of the cohesive force unit in the normal and tangential directions, respectively; and η is a material parameter. The parameters for the mortar–aggregate cohesive element [34] are provided in Table 3.

**Figure 7:**
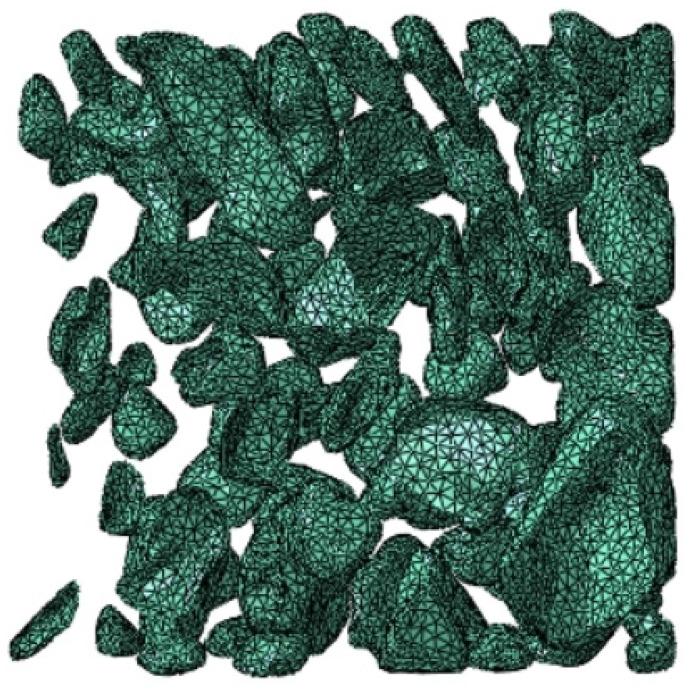
Cross-sectional view illustrating cohesive elements embedded at mortar–aggregate interface.

The mortar–aggregate interface in the mesoscopic ASSCC model was modeled with shared nodes. Therefore, zero-thickness cohesive elements were directly incorporated at the mortar–aggregate interface in ABAQUS. These cohesive elements were defined as shell elements with null volume, as illustrated in Figure 7.

#### 3.2.3. Application of Loading and Definition of Boundary Conditions

**Figure 8:**
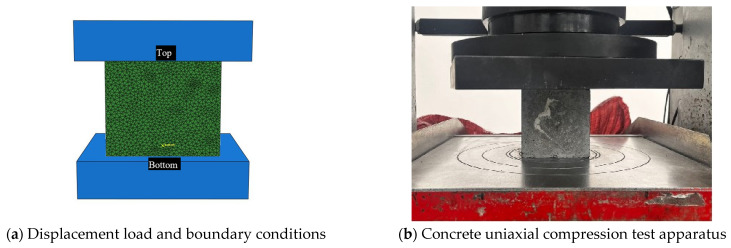
Loading schematic for numerical simulation and experimental test of ASSCC under uniaxial compression.

The mesoscopic ASSCC model was configured as follows: A uniform displacement load was applied to the top surface of the specimen, while the bottom surface was subjected to a fixed constraint. Both the loading platen and the base platen were defined as rigid bodies. A frictional contact with a friction coefficient of 0.2 was established between the concrete specimen and the rigid platens, as illustrated in Figure 8.

### 3.3. Validation of the Mesoscopic Model

#### 3.3.1. Mesh Sensitivity Analysis

To address the mesh sensitivity issue in the mesoscopic ASSCC model, models with mesh sizes of 1 mm, 2 mm, 3 mm, and 4 mm were established while retaining the geometry of the concrete phases. These models were subjected to uniaxial compression simulations.

**Figure 9:**
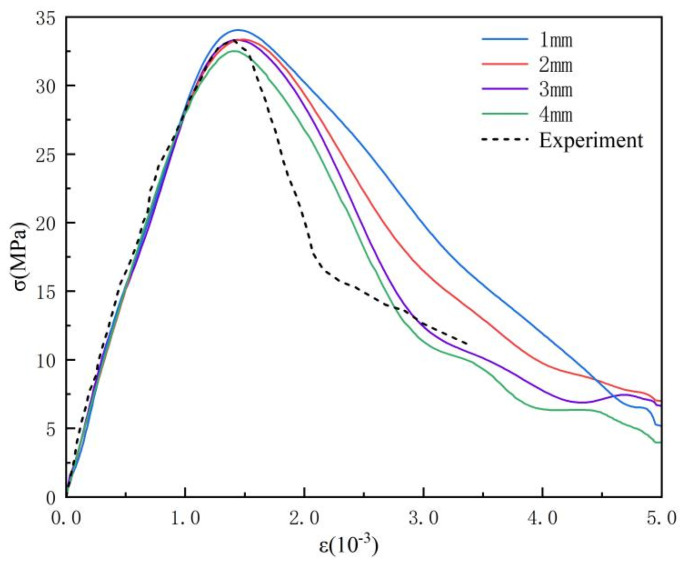
Stress–strain curves for models with mesh sizes varying from 1 mm to 4 mm.

Figure 9 depicts that the stress–strain curves obtained from the four models with different mesh sizes exhibited good agreement with the experimental curve during the elastic stage. The peak stresses for the models with mesh sizes of 2 mm and 3 mm were 33.36 MPa and 33.31 MPa, respectively, both of which aligned well with the peak stress of the experimental curve. In contrast, the peak stresses for the models with mesh sizes of 1 mm and 4 mm deviated significantly from the experimental data. This finding suggests that both excessively fine and excessively coarse mesh sizes introduce computational errors. A finer mesh enables a more detailed description of progressive interface damage and stress concentration, leading to a higher predicted compressive strength. In contrast, a coarser mesh results in premature and overly extensive interface failure, yielding a lower compressive strength. Regarding the descending branch, the stress–strain curves predicted by models with varying mesh sizes exhibit generally poor agreement with the experimental curve. This discrepancy is mainly attributable to the simplifications adopted in the mechanical parameters of the individual concrete phases, coupled with the inherent inability of the CDP damage model to fully replicate the complex damage evolution behavior of the material during the post-peak softening regime. Ultimately, a mesoscopic ASSCC model with a mesh size of 3 mm was selected for further analysis. This configuration contained approximately 1,002,000 tetrahedral elements (C3D4) and 163,000 nodes.

#### 3.3.2. Numerical Simulation Results Under Uniaxial Compression

**Figure 10:**
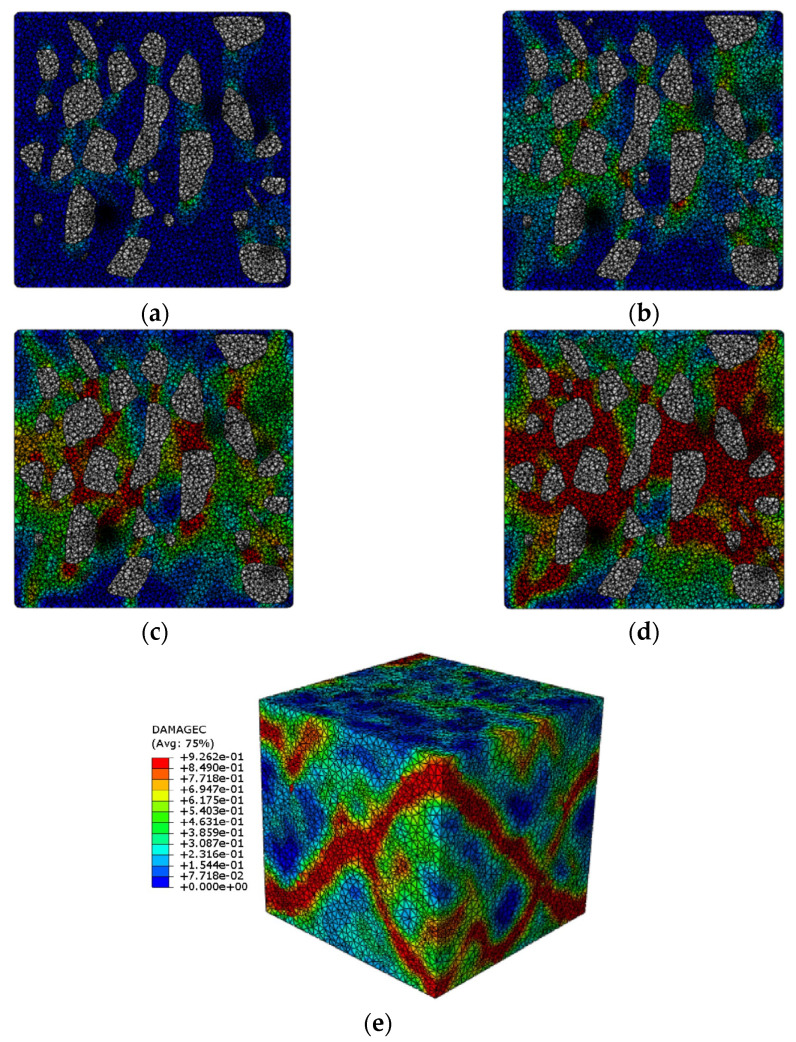
Failure process of ASSCC under uniaxial compression: (**a**) initial loading stage; (**b**) intermediate loading stage; (**c**) ultimate bearing capacity; (**d**) post-peak loading stage; (**e**) final damage pattern.

The numerical results of the uniaxial compression tests on ASSCC are presented in Figure 10. During the initial loading stage, the ITZ between the aggregate and mortar acted as a stress concentration zone due to its relatively lower stiffness, particularly near the sharp edges of the aggregates (Figure 10a). Increasing stress level led to the initiation of microcracks within the ITZ (Figure 10b). The propagation and coalescence of these microcracks led to the formation of macroscopic cracks that contributed to the failure of some elements. Consequently, the ASSCC entered the softening stage (Figure 10c). Ultimately, multiple major cracks coalesced to form an “X”-shaped failure surface, which marked the complete loss of load-bearing capacity and the structural failure of the ASSCC (Figure 10d). A comparison between the experimental and simulated final compressive failure patterns (Figure 10e) reveals that both exhibit an “X”-shaped failure surface with microcracks of varying degrees of damage on the surface, validating that this three-dimensional mesoscale model can essentially simulate the uniaxial compressive damage process of ASSCC.

## 4. Results and Analysis

### 4.1. Impact of Freeze–Thaw Cycles on the Pore Structure of ASSCC

**Figure 11:**
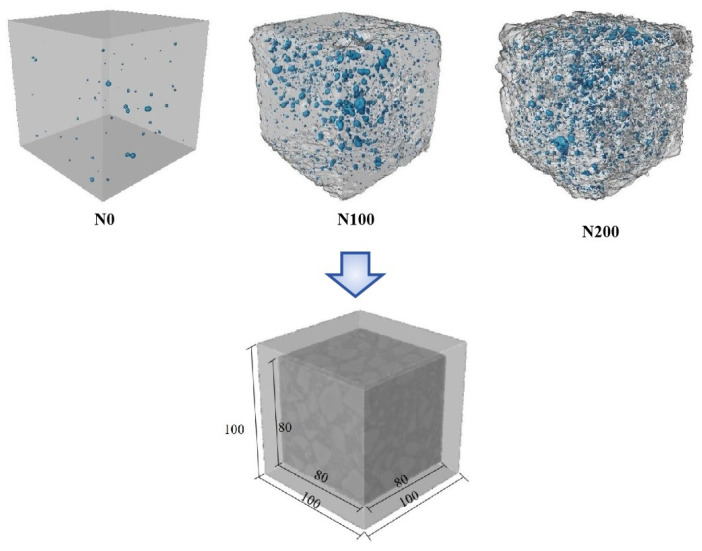
Pore structure of ASSCC-S20 specimen under different freeze–thaw cycles (unit: mm).

Based on CT scan data, the pore structures of ASSCC specimens after 0, 100, and 200 freeze–thaw cycles were characterized via threshold segmentation. It should be noted, however, that the present analysis is limited to a single representative specimen, which constrains the statistical generalizability of the observations. As the number of freeze–thaw cycles increases, the internal pore structure of ASSCC gradually becomes more complex, with pores expanding and interconnecting to form microcracks. This leads to the spalling of surface mortar and the loss of some irregular pores, thereby affecting the accuracy of pore structure analysis. To address this, an 80 mm × 80 mm × 80 mm central region of the concrete was selected (see Figure 11). By analyzing changes in planar porosity, equivalent pore diameter, and morphological characteristics, the degradation mechanism of pore structure under freeze–thaw action was investigated.

#### 4.1.1. Planar Porosity

**Figure 12:**
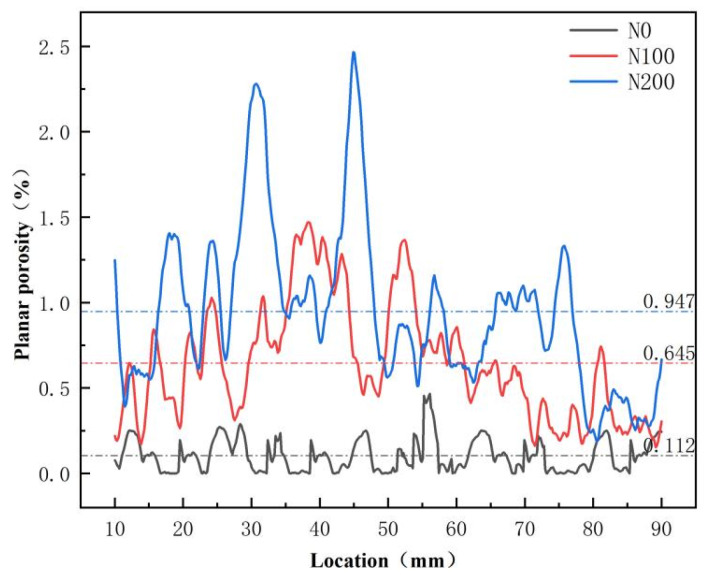
Planar porosity of ASSCC specimens under different freeze–thaw cycles.

Figure 12 revealed that ASSCC specimen without experiencing freeze–thaw cycles displayed a uniform pore distribution and a low planar porosity of 0.112%. This can be attributed to the synergistic enhancement of matrix density resulting from the high fluidity of SCC and the incorporation of aeolian sand. However, as the freeze–thaw cycles increased, the porosity increased significantly. During the initial stage (0–100 N_f_), rapid pore formation and interconnection led to an increase in porosity to 0.645%. During the later stage (100–200 N_f_), the pore formation rate declined. After 200 freeze–thaw cycles, the porosity reached 0.947%. The results indicate that after 100 freeze–thaw cycles, the overall porosity increase is the dominant feature of the ASSCC material. After 200 cycles, however, the dominant feature shifts to the local connectivity of the pore structure.

#### 4.1.2. Equivalent Pore Diameter

The equivalent diameter is a key parameter for characterizing the pore size distribution and serves as a primary metric for describing pore dimensions. It is defined as the diameter of a circle with the same area as the pore cross-section observed in a two-dimensional image. The calculation formula is as follows:

(5) $$D_{e}=\sqrt[{3}]{\frac{6V}{\pi }}$$

where *D_e_* is the equivalent spherical diameter; *V* is the pore volume; and *π* is the mathematical constant Pi.

**Figure 13:**
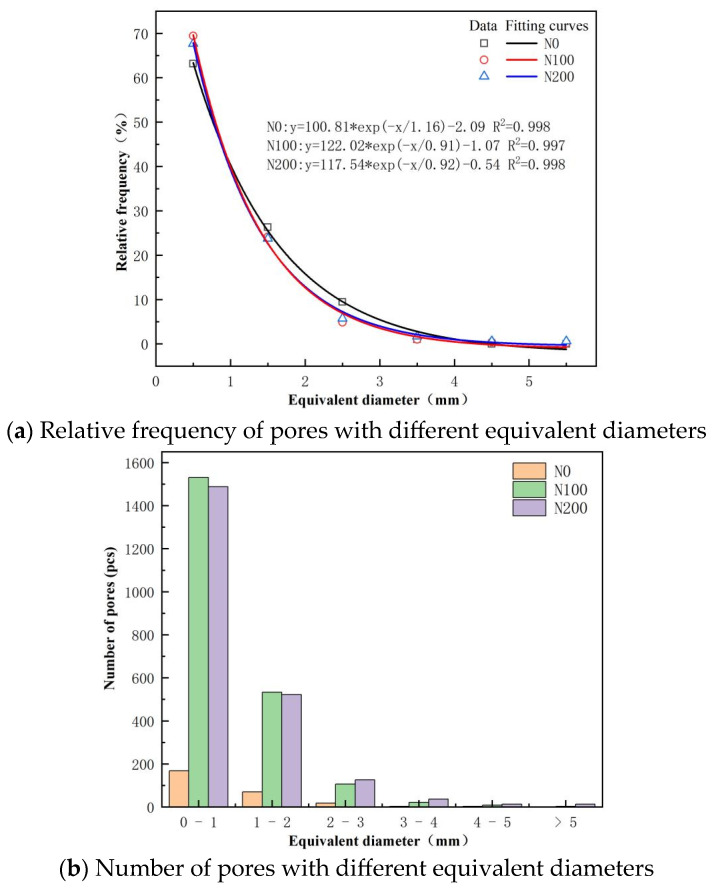
Variation in equivalent pore diameters under different freeze–thaw cycles.

Figure 13a shows that the equivalent pore diameters of ASSCC specimens under varying freeze–thaw cycles followed an exponential distribution and were predominantly distributed within the range of 0–1 mm. Figure 13b shows that the untreated ASSCC specimen (0 Nf) contained a limited number of pores. The number of pores increased dramatically after 100 freeze–thaw cycles. Specifically, pores with equivalent diameters in the 0–1.0 mm range were 7.5 times those in the untreated specimen, while those in the 1.0–2.0 mm range increased by 6.6 times. The number of pores in the 0–2.0 mm range slightly decreased after 200 freeze–thaw cycles compared to the specimen subjected to 100 freeze–thaw cycles. However, the number of pores larger than 2 mm increased.

In summary, during the first 100 freeze–thaw cycles, numerous small pores developed within the ASSCC specimens. As the number of cycles increased, adjacent small pores gradually interconnected and coalesced to form larger pores. Between 100 and 200 cycles, the pore evolution transitioned into a slower development stage, during which pore growth occurred primarily through the interconnection and merging of existing small pores.

#### 4.1.3. Pore Sphericity

**Figure 14:**
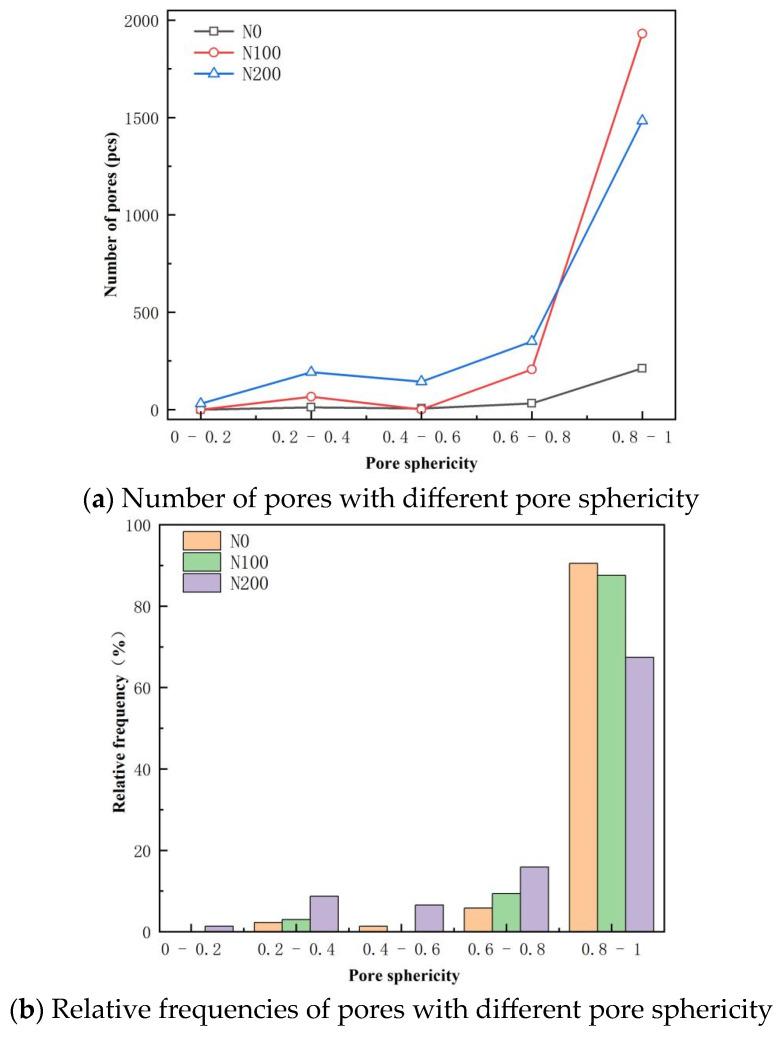
Pore sphericity distribution of ASSCC under different numbers of freeze–thaw cycles.

Concrete strength is influenced not only by porosity and pore size, but also significantly by pore morphology. Among these factors, pore sphericity—a geometric parameter quantifying how closely a pore resembles an ideal sphere, with values ranging from 0 to 1—serves as one of the key controlling factors directly affecting the mechanical properties of the material. A sphericity value closer to 1 indicates a more spherical and regular pore morphology. Pore sphericity is closely related to the frost resistance of concrete. Lower sphericity indicates a more irregular pore shape, which is more prone to deformation during freeze–thaw cycles, thereby reducing the frost resistance of the concrete. Therefore, investigating the distribution of pore sphericity within ASSCC under different freeze–thaw cycles is of great importance. The distribution of pore sphericity is presented in Figure 14.

As can be seen from Figure 14, the pore sphericity of unfrozen ASSCC is predominantly distributed between 0.8 and 1.0, indicating relatively regular pore shapes and a limited number of pores. After 100 freeze–thaw cycles, the number of pores increases significantly. Although the newly formed pores remain relatively regular in shape, the relative frequency of pore sphericity deviating from 1 shows a slight increase, reflecting the initial damage induced by freeze–thaw action on ASSCC. Following 200 freeze–thaw cycles, the relative frequency of pore sphericity deviating from 1 rises markedly. The number of pores with sphericity between 0.8 and 1.0 decreases by 26.3% compared to that after 100 cycles, while the number of pores with sphericity in the range of 0–0.8 increases by 161.3%. This trend can be attributed to the progressive complication of the internal pore structure under continuous freeze–thaw cycling, during which pores undergo continuous expansion and interconnection, leading to the formation of microcracks. Consequently, the internal matrix of the concrete becomes loosened, resulting in a reduction in mechanical strength.

#### 4.1.4. Evolution of Pore Structure in ASSCC Under Freeze–Thaw Action

Based on quantitative analysis of mesoscopic parameters from CT scan images, including planar porosity, pore equivalent diameter, and pore sphericity, the results indicate that ASSCC exhibits typical staged characteristics of pore evolution under freeze–thaw cycles: Initial stage (0–100 Nf): Porosity increases sharply, dominated by regular small pores. Later stage (100–200 Nf): Pore connectivity accelerates, forming irregular large pores, with significant deterioration in pore sphericity and concomitant microcrack propagation. This reveals that the freeze–thaw damage mechanism originates from a dynamic evolution process involving pore quantity growth → connectivity → morphological complexity.

### 4.2. Numerical Simulation of the Freeze–Thaw Cycles in Mesoscopic Model of ASSCC

Existing studies [5,7] generally concur that the primary damage in concrete during freeze–thaw cycles occurs predominantly during the freezing phase. Therefore, it can be assumed that the damage mainly occurs during the freezing, ignoring the damage during the thawing phase [22]. A unidirectional freezing load (a non-cyclic process) was applied to the mesoscopic model. The freeze–thaw damage mechanism was modeled by assigning expansion properties to pore elements in response to temperature decrease.

#### 4.2.1. Determination of the Number of Freeze–Thaw Cycles

**Figure 15:**
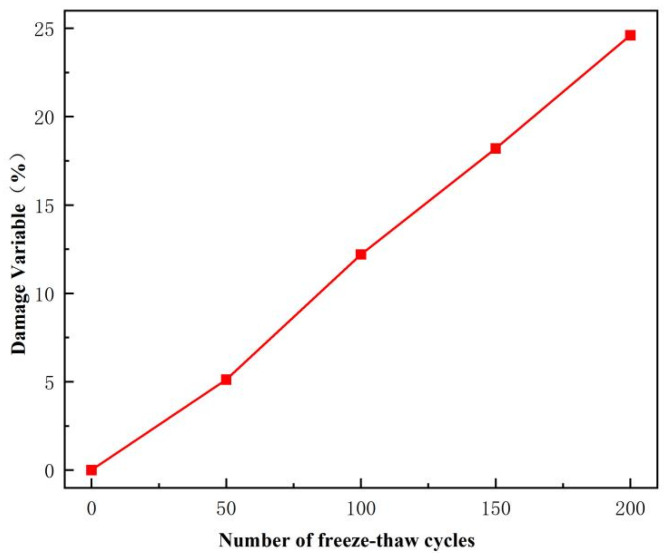
Damage degree of ASSCC under different freeze–thaw cycles.

Since a non-cyclic freezing load was applied to the mesoscopic ASSCC model in ABAQUS, it is not possible to directly correlate the simulation results with a specific freeze–thaw cycle. Therefore, it is necessary to establish a relationship between the freeze–thaw cycles and the numerical simulation results of freezing damage in ASSCC. The damage variable is a commonly used dimensionless parameter for quantifying the development of internal micro-defects (such as microcracks and pores) in concrete materials. It provides a measure of the degradation in mechanical properties caused by loading or environmental factors. Common indicators used to characterize concrete damage include the loss rates of elastic modulus, stress, strength, and energy dissipation. This study employed the elastic modulus to characterize the damage in ASSCC during freeze–thaw cycles. The freeze–thaw cycles corresponding to the numerical simulation results in ABAQUS were determined based on the damage degree. The experimental damage degree is illustrated in Figure 15. Since the elastic modulus cannot be directly measured in experiments, the relative dynamic elastic modulus was used instead to calculate the damage degree of ASSCC during freeze–thaw cycles. This is achieved using Equation (6):

(6) $$D\left(n\right)=1-\frac{P\left(n\right)}{P\left(0\right)}$$

where *D*(*n*) is the damage variable after n freeze–thaw cycles; *P*(*n*) is the relative dynamic elastic modulus of concrete after n freeze–thaw cycles; and *P*(0) is the initial relative dynamic elastic modulus of concrete before freeze–thaw cycles.

#### 4.2.2. Numerical Simulation Results of Freeze–Thaw Cycles

**Figure 16:**
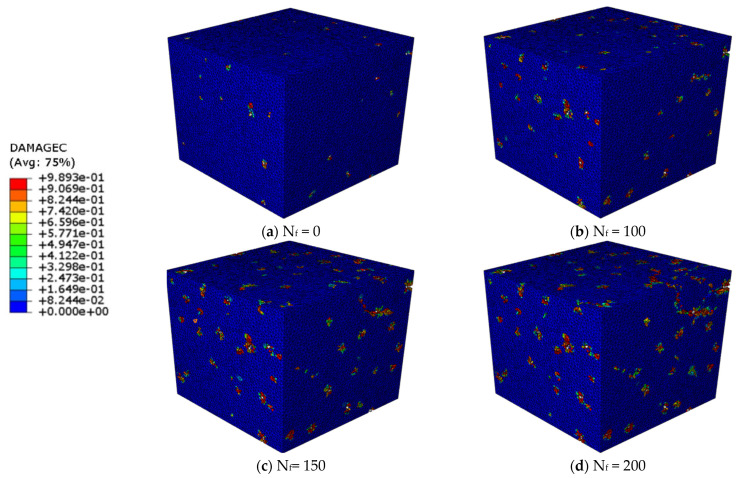
Damage morphology of ASSCC under different freeze–thaw cycles.

The damage morphology of ASSCC under different freeze–thaw cycles is illustrated in Figure 16.

After 50 freeze–thaw cycles, pronounced stress concentration was observed on the surface of the concrete specimen. Localized elements were deleted as the accumulation of damage reached the failure threshold. After 100 freeze–thaw cycles, stress concentration zones on the concrete model surface increased, and localized spalling notches formed in the edge regions. At 150 freeze–thaw cycles, micro-pores on the surface of the concrete specimen expanded and interconnected to form initial microcracks, which marked the onset of an accelerated development stage of freeze–thaw damage. When the freeze–thaw cycles increased to 200, cracks propagated along stress concentration paths and connected with adjacent damaged zones, ultimately leading to the formation of macroscopic primary cracks.

**Figure 17:**
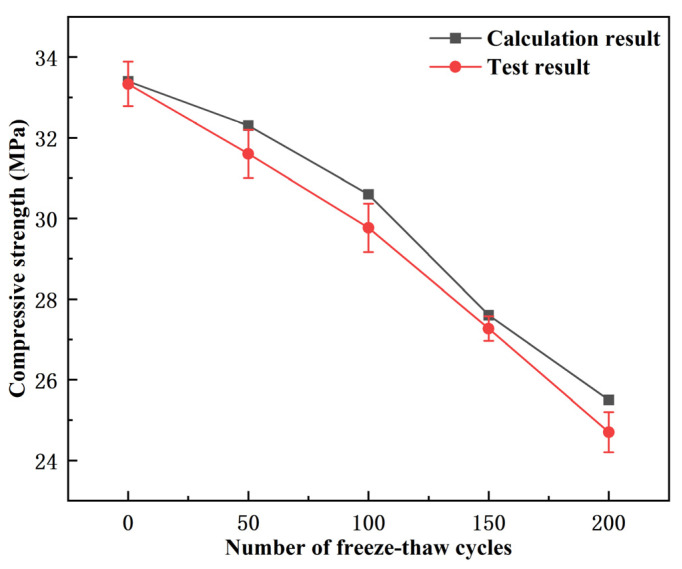
Variation in numerical and experimental compressive strengths of ASSCC.

Restart analysis was performed on the mesoscopic models of ASSCC subjected to different numbers of freeze–thaw cycles, followed by uniaxial compression simulations. As shown in Figure 17, after 50, 100, 150, and 200 freeze–thaw cycles, the mean compressive strength of ASSCC decreased by 5.1%, 10.8%, 18.9%, and 25.8%, respectively. The compressive strength exhibited a significant cumulative damage trend with increasing freeze–thaw cycles, with a more pronounced cumulative effect in the later stages compared to the initial phase. The numerical simulation results of uniaxial compressive strength showed a relative error of less than 3% compared to experimental data, indicating that the simulation accurately captured the influence of freeze–thaw damage on the mechanical properties of concrete. This study provides a theoretical basis for the durability assessment of aeolian sand self-compacting concrete under freeze–thaw environments.

## 5. Conclusions

Based on X-ray computed tomography (CT) scanning technology, two-dimensional slices of aeolian sand self-compacting concrete (ASSCC) were obtained. The geometric models of distinct material phases were extracted using image processing software, subsequently optimized and assembled in 3-matic software. The reconstructed mesoscopic model of ASSCC was ultimately imported into ABAQUS software for uniaxial compression and freezing simulations. The results indicated:

(1) Characteristics of Pore Evolution in ASSCC under Freeze–Thaw Cycles: Initial Stage (0–100 N_f_): Porosity increases rapidly, dominated by regularly shaped small pores with relatively uniform morphology and small equivalent diameters. Later Stage (100–200 Nf): Pores progressively connect and coalesce, forming irregular large pores accompanied by microcrack propagation. Pore sphericity deteriorates significantly, revealing that the freeze–thaw damage mechanism stems from a dynamic evolution process involving pore quantity growth → connectivity → morphological complexity.
(2) A 3D geometric model of mortar, aggregate, and pores was reconstructed by processing the raw CT images using image analysis software. The established mesoscale model of ASSCC accounts for the influence of the ITZ and mesh element size on the accuracy of simulation results, thereby achieving improved modeling fidelity. Unlike conventional random aggregate concrete models [22,23], the proposed CT-based reconstruction method retains the actual aggregate and pore morphology, which leads to more realistic stress concentration predictions.
(3) Under uniaxial compression, the failure of ASSCC initiates with the nucleation of microcracks in the ITZ, which subsequently propagate along the aggregates, ultimately leading to the formation of an X-shaped macroscopic fracture and a loss of bearing capacity. The experimental and numerical results show good agreement, validating the reliability of the mesoscale model in simulating the damage process of ASSCC.
(4) A non-cyclic freezing numerical simulation was performed on the mesoscopic ASSCC model. The freeze–thaw cycles corresponding to various damage morphologies were determined based on the damage degree. The compressive strength of ASSCC under 0–200 freeze–thaw cycles was obtained, and the error in compressive strength between the simulated and experimental results was within 3%, demonstrating the reliability of the numerical simulation.

## Figures and Tables

**Table 1:** Mix proportions.

Mixture ID	Mix Proportions of Concrete per Cubic Meter by Mass (kg/m^3^)						
	Cement	Fly Ash	Water	Aeolian Sand	Natural Sand	Coarse Aggregate	Superplasticizer
ASSCC	259.6	258.1	173	140.8	565.5	851	3.5

**Table 2:** Mechanical parameters of aggregate and mortar materials.

Material	Elastic Modulus (GPa)	Poisson’s Ratio	Compressive Strength (MPa)	Tensile Strength (MPa)
Aggregate	82.8	0.35	/	/
Mortar	21.2	0.2	32	3.4

**Table 3:** Parameters for the mortar–aggregate cohesive element.

Mode I Tensile Strength (MPa)	Mode II Shear Strength (MPa)	Mode I Fracture Energy (N/mm)	Mode II Fracture Energy (N/mm)	η
2.6	10	0.025	0.625	1.2

## Data Availability

The original contributions presented in the study are included in the article; further inquiries can be directed to the corresponding author.
